# The structure of deviations from maximum parsimony for densely-sampled data and applications for clade support estimation

**DOI:** 10.1109/TCBBIO.2025.3595782

**Published:** 2025

**Authors:** William Howard-Snyder, Will Dumm, Mary Barker, Ognian Milanov, Claris Winston, David H Rich, Marc A Suchard, Frederick A Matsen

**Affiliations:** 1 Department of Computer Science and Engineering, University of Washington, Seattle, WA; 2 Computational Biology Program, Fred Hutchinson Cancer Research Center, Seattle, WA; 3 Department of Computer Science, Princeton University, Princeton, NJ; 4 Department of Human Genetics, University of California, Los Angeles; 5 Department of Computational Medicine, University of California, Los Angeles; 6 Department of Biostatistics, University of California, Los Angeles; 7 Howard Hughes Medical Institute, Seattle, WA; 8 Department of Genome Sciences, University of Washington, Seattle, WA; 9 Department of Statistics, University of Washington, Seattle, WA

**Keywords:** Phylogenetics, Maximum Parsimony, Directed Acyclic Graph

## Abstract

*How* do phylogenetic reconstruction algorithms go astray when they return incorrect trees? This simple question has not been answered in detail, even for maximum parsimony (MP), the simplest phylogenetic criterion. Understanding MP has recently gained relevance in the regime of extremely dense sampling, where each virus sample commonly differs by zero or one mutation from another previously sampled virus. Although recent research shows that evolutionary histories in this regime are close to being maximally parsimonious, the structure of their deviations from MP is not yet understood. In this paper, we develop algorithms to understand how the correct tree deviates from being MP in the densely sampled case. By applying these algorithms to simulations that realistically mimic the evolution of SARS-CoV-2, we find that simulated trees frequently only deviate from maximally parsimonious trees locally, through simple structures consisting of the same mutation appearing independently on sister branches. We leverage this insight to design approaches for sampling near-MP trees and using them to efficiently estimate clade supports.

## I. Introduction

Although there have been hundreds of papers documenting the performance of phylogenetic algorithms, it can be difficult to understand *why* phylogenetic algorithms do not succeed when they come up short. Even for maximum parsimony (MP), the simplest phylogenetic criterion, the multitudes of equally parsimonious trees complicates assessment of error, because that phylogenetic error may depend substantially on the arbitrary choice of which MP tree to report. Understanding MP has recently gained relevance in the regime of extremely dense sampling, in which each virus sample is commonly zero or one mutation away from another previously sampled virus. Recent research has shown that the generating trees for data sets in this regime are close to being maximally parsimonious using theoretical [1] and empirical [2] analysis. However, the structure of these deviations from MP is not yet understood.

In this paper, we develop algorithms to understand the structure of deviations from maximum parsimony (MP) in simulations that realistically mimic the difficulty of phylogenetic reconstruction for SARS-CoV-2, and use this understanding to accurately estimate clade support from MP trees. To do so, we introduce a novel summary statistic, *parsimony diversity*, which enables us to generate data sets with realistic inferential difficulty. We then develop algorithms to find the nearest MP tree among the many possible MP trees to the simulated tree, and compare the structure of this tree and its associated mutations to the simulated tree. We find that differences between simulated trees and their most similar MP counterparts primarily consist of simple structures involving parallel child mutations on sister branches. We explore one application of this observation to estimate clade support, a measure of confidence in particular groupings of observed taxa in the inferred tree, by sampling MP trees and randomly injecting subparsimonious structure. Experiments on simulated data demonstrate that our support estimator yields more accurate support for simulated densely-sampled data than traditional approaches like the phylogenetic bootstrap or Bayesian clade support using existing implementations.

**Fig. 1:**
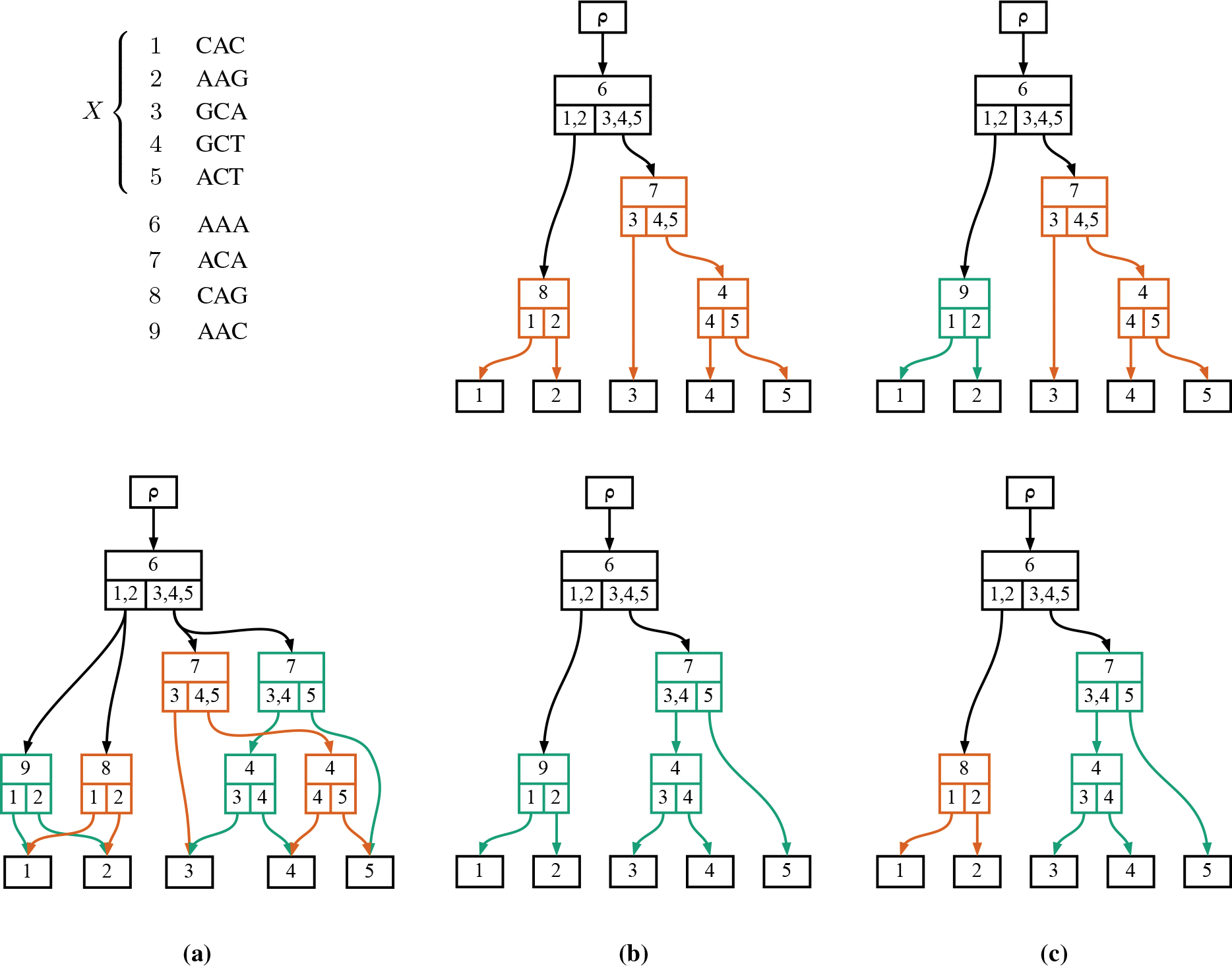
A sequence alignment X and a history sDAG containing four histories relating the sequences of X (a). Columns (b) and (c) show all histories contained in the history sDAG. A “history” is a tree structure such that each node consists of a label ℓ (shown on the top of each node), and a child clade set U, a set of sets of leaf node labels reachable from the node’s children (shown on the bottom half of each node). The history sDAG is a graph union of the two histories in (b), allowing the green and orange subtrees to be swapped, creating the histories in (c). The top of each node shows the node label, which is a sequence, while the bottom shows child clade sets, with edges drawn to exit the child clade set which matches the target node’s clade. Colors illustrate alternative subtrees, and are not part of the data structure.

The steps of this procedure would not be possible without our recent development of a means of compactly representing highly parsimonious trees called the “history sDAG” (Figure 1). Building on the matOptimize software [3], we have developed Larch [4], an open-source software that can search for diverse MP trees on data and produce a history sDAG containing these trees. Using the history sDAG and this software, we are often able to find well over 10^10^ highly parsimonious trees for SARS-CoV-2 data sets, even when we collapse branches without mutations and restrict to trees that are as parsimonious as any that the phylogenetic algorithm has found [5]. By enabling us to enumerate potentially all MP trees on simulated data, the history sDAG allows an efficient estimation of parsimony diversity, our summary statistic for comparing the difficulty of phylogenetic inference on simulated and real data. The structure of the history sDAG containing all MP trees on a dataset facilitates dynamic programming algorithms that can rapidly find the closest MP tree to a given simulated tree, even when the number of MP trees is beyond astronomical. The history sDAG also enables efficient sampling from this large set of MP trees. In this paper, we show how uniform sampling from the trees in the history sDAG combined with a principled perturbation produces high accuracy clade support estimates.

## II. Methods

### A. The history sDAG

A key component of our analysis is a structure called the history sDAG. We present an overview of the history sDAG here, but for further details, please refer to [5]. The history sDAG enables efficient storage of many phylogenetic histories, which are phylogenetic trees that include additional identifying data on internal nodes, such as inferred ancestral sequences. In addition to its storage efficiency, this data structure has useful properties when storing MP histories.

When one constructs a history sDAG from a limited number of MP histories, the resulting structure often includes extra histories, and these are guaranteed to also be MP. These new histories arise from swapping subhistories between the input histories, in a manner that preserves parsimony score. This capability makes the history sDAG valuable for exploring and summarizing the landscape of MP histories in a dataset. In this study, we use the history sDAG to store a collection of MP histories efficiently on simulated data and to swiftly identify the MP history most similar to the simulated history.

The history sDAG is simply a graph union of histories, augmented with redundant node information so that individual histories can be easily identified within the structure of the history sDAG. Specifically, histories are defined so that the information augmented to each node consists not only of the observed and ancestral sequence data, but also the *subpartition* of the node’s clade formed by the clades of the node’s children. Figure 1 shows an example of a simple history sDAG and the four histories it contains.

In preparation for Definition 1, which formalizes this information and the notion of histories in this paper, let X be a set of aligned nucleotide sequences, and let (V,E) be a directed graph with a *universal ancestor* (*UA*) *node* denoted ρ∈V, and with the edge set E⊂V×V. For each node v∈V\{ρ}, let v be a pair (ℓ,U). The sequence ℓ and the set U are referred to as the *label* and *child clade set* of v, respectively. If v is a leaf node, ℓ∈X and U=∅. Let C(v)⊂X be the *child clade* of v, consisting of the set of labels of leaf nodes reachable from v.

#### Definition 1.

*A directed acyclic graph* (V,E) *with a universal ancestor node is a history if it is tree-shaped, and if for any non-leaf node* v=(ℓ,U)∈V,U *is such that*

$$U=\left\{C\left(v_{c}\right)|v_{c}\in Ch(v)\right\},$$

*where* Ch(v) *are the children of* v.

*A structure* ($V^{\prime },E^{\prime }$) *is a* sub-history *of* (V,E) *if* $V^{\prime }\subset V$ *and* $E^{\prime }\subset E$, *and these sets contain exactly one node* v∈V *and all nodes and edges reachable from* v *in* (V,E).

Given this definition of a history, we can now concisely define the history sDAG.

#### Definition 2.

*Let* T *be a set of histories, and define*

$$V^{\prime }\;=\underset{(V,E)\in T}{⋃}V\;\mathrm{and}\;E^{\prime }\;=\underset{(V,E)\in T}{⋃}E.$$

*Then* ($V^{\prime },{\;E}^{\prime }$) *is a* history sDAG. *We may also say that* ($V^{\prime },E^{\prime }$) *is the history sDAG* constructed from T. *For any* $V_{t}\subset V^{\prime }$ *and* $E_{t}\subset E^{\prime }$ *such that* ($V_{t},E_{t}$) *is a history, then we say that the history sDAG* ($V^{\prime },E^{\prime }$) contains *the history* $\left(V_{t},E_{t}\right)$.

Since the structure of a history determines the child clade set of each node, the definition of a history implicitly guarantees that edges have additional properties. Notice that for any edge e∈E in a history (V,E), if $e=\left((\ell ,U),v_{c}\right)$, then $C\left(v_{c}\right)\in U$. Furthermore, for each node v=(ℓ,U)∈V, and for each child clade C∈U, there must be exactly one edge $\left(v,v_{c}\right)\in E$ such that $C\left(v_{c}\right)=C$. Additional edges descending from v with this property cannot exist. If they did, leaves with labels in C would be reachable by multiple paths, which is impossible since a history is a tree. This is an important property of edges in a history that allows us to recover individual histories from the history sDAG.

To sample a history from a history sDAG, one need only choose an edge descending from the UA node ρ, then choose one edge descending from each child clade of each node of the chosen edge’s target node. By continuing this process until the leaf nodes, which have no child clades, one obtains a history contained in the history sDAG. In Figure 1, edges are drawn exiting their parent node from the child clade that matches their target node’s clade, and each history in Figure 1 can be seen as the result of this process of iteratively choosing edges starting from the UA node of the history sDAG. We can sample uniformly from the collection of histories in the history sDAG by weighting our choice of edge descending from each clade proportional to the number of subtrees possible below the edge’s target node.

### B. Minimum weight trimming

As a combinatorial object, the history sDAG can easily contain a vast number of histories, typically combinatorially larger than the set of histories from which it is constructed. These additional histories result from swapping subhistories between the input histories, a simple example of which Figure 1 shows. In previous work [5], we showed how it is not uncommon for the history sDAG on real data to have 8 orders of magnitude more histories than the number of histories used to build it.

Conveniently, the structure of the history sDAG offers an efficient way of computing certain weights on those histories, and identifying the subset of histories which maximize or minimize such a weight. We refer to this process of identifying optimal histories with respect to a weight as *trimming* the history sDAG. In [5], we describe a dynamic programming algorithm to trim the history sDAG so that it expresses only its minimum weight histories. The algorithm involves removing all edges which point to suboptimal subhistories, and can be realized in two traversals of the history sDAG. We define that algorithm in more detail below.

This efficient trimming algorithm can be applied for any history weight whose value decomposes as a sum over edges in each history. More precisely, let T be the set of all histories contained in history sDAG(V,E), and let g:T→R be a function which assigns weights to histories. For any history t∈E, let the set of edges in t be denoted $E_{t}$. The trimming algorithm can be used if there exists an edge weight function f:E→R so that $g(t)=\sum_{e\in E_{t}}f(e)$, for any history t∈T.

Given such a history weight, we can compute the minimum weight of any subhistory beneath a node v∈V, which we will denote $M_{f}(v)$, in a single post-order traversal of the history sDAG [5]. Denote this operation **AnnotateMinWeight**(V,E). The full trimming algorithm is presented in the Supplementary Materials (Section V-B), and involves removing edges from the history sDAG which do not minimize subhistory weight below a node. The output of this algorithm, which we will call **MinTrim**(V,E,f), is a new history sDAG that contains only those histories in the history sDAG (V,E) that minimize the weight function g.

This trimming algorithm is essential for our methods in two ways. First, it allows us to trim the history sDAG to contain only histories that minimize the parsimony score, which is a sum of mutations on each edge in a history. We also use this algorithm to trim a history sDAG containing MP histories to contain only histories minimizing Robinson-Foulds (RF) distance to a fixed reference history. In the Supplementary Material (Section V-A) we describe how RF distance can be decomposed as a sum over edges, making this application of the algorithm possible.

### C. MP-history search with the history sDAG

Many of our algorithms depend on producing and efficiently storing a large collection of MP histories. We describe this subroutine here. Given a set of aligned sequences X and a number of iterations n, we execute the following steps:

1. Build a tree from the sequences in X (with UShER) by greedily placing leaves one at a time so as to minimize the parsimony score.
2. Build a history sDAG (V,E) from that tree.
3. Repeat the following steps n times.
  - Sample a history t uniformly from (V,E).
  - Greedily select SPR moves (with Larch) to minimize the parsimony score of t.
  - Graph union the result to (V,E).
4. Return **MinTrim**(V,E,f) where f:E→Z counts the number of mutations on edge e.

Sampling a history in step 3a and adding it to the history sDAG in step 3c take time proportional to the number of nodes, which is O(X). Optimizing the history greedily in step 3b takes $O\left(X^{2}\;log\;X\right)$ in the worst case, but Larch caches Fitch sets and uses parallelism to make it run very fast in practice. The overall runtime of this algorithm is therefore $O\left(E+nX^{2}\;log\;X\right)$ and the space complexity is O(E+V). In our experiments, we use n=20,000. We refer to the trees contained in the trimmed history sDAG as *inferred-MP histories*. Recall that although we may only add up to n histories to the history sDAG, the number of histories discovered is often much larger than n due to subhistory swapping. This allows our algorithm to find many orders of magnitude of inferred-MP histories, while maintaining space and memory efficiency. For instance, this procedure found over 10^20^ inferred-MP histories on the UShER-clade A.2.5 with 620 unique sequences.

Steps 2 and 3 are implemented in Larch, an open-source C++ implementation of the history sDAG at https://github.com/matsengrp/larch. We implemented step 4 using the Python implementation of the history sDAG at https://matsengrp.github.io/historydag. All bash scripts, Python experiments, code for generating plots, and instructions on how to reproduce results can be found in our GitHub repository at https://github.com/matsengrp/hdag-benchmark.

The MP trees we attempt to find, and those stored in the resulting history sDAG are *collapsed*, in the sense that all non-pendant edges must have mutations on them. We make this choice to avoid storing nodes which result from arbitrary binary resolutions of clades, when there is no signal in the sequence data to support a particular resolution. Therefore, the true number of binary MP trees is much larger than the number of MP trees stored in the history sDAG. Supposing that all collapsed MP histories were contained in the history sDAG however, any binary MP history could be realized as a resolution of a collapsed history, with resolved edges containing no mutations.

### D. Parsimony diversity

Real sequence data often support many phylogenetic trees, making it challenging to accurately infer the true generating tree. Given that we want our claims about the simulated data to generalize to real data, we ensure that phylogenetic inference on the simulated data is sufficiently difficult. We use Parsimony Diversity (PD) as a proxy for how hard an inference problem is. Parsimony Diversity is defined as *the number of unique clades among fully-collapsed MP trees*. Under the MP criterion, these are the groupings of taxa that the data supports. Additionally, in the densely sampled regime, the MP criterion and ML criterion converge [1], so the clades that appear in MP trees should be a reasonable summary of the most likely clades in our setting. Previous work shows that similar summary statistics measuring the diversity of MP trees are the most important predictors of the difficulty of phylogenetic inference [6]. In the sections below, we describe how we use Parsimony Diversity to tune the inferential difficulty of our simulated datasets.

#### Simulations:

Following previous work [7], we start with the background tree on over 2 million SARS-CoV-2 genomes at http://hgdownload.soe.ucsc.edu/goldenPath/wuhCor1/UShER_SARS-CoV-2/2022/10/01 [8] and inferred using UShER [3]. We first select subtrees containing between 500 and 1000 (non-unique) leaves. We must also fully resolve these subtrees because directly simulating on the UShER subtrees would create too many multifurcations from edges without mutations. Then, for each subtree we:

1. Randomly resolve all multifurcations and give new edges small branch lengths of 1/1000th the length of a branch with a single mutation.
2. Use phastSim [9] to simulate sequences on the tree starting with the Wuhan-1 reference sequence. We repeat this step until every leaf sequence forms a monophyletic clade with its duplicate sequences.
3. Retain a subset of the leaves so that leaves are uniquely labeled by their sequences.
4. Write an alignment containing all the unique leaf sequences in this tree.

In step 2, we simulate using the UNREST substitution model with empirically determined rates, Gamma distributed rate variation, and hypermutation. On average, the total tree length was 0.032 and the substitution rate per site was 1. We include hypermutation to better fit the patterns of mutability observed in SARS-CoV-2, which indicate that some sites have much larger base-to-base mutation rate than others, possibly reflecting selective pressure [10]. PhastSim [9] models this effect by setting sites as hypermutable with probability p and scaling a randomly selected base-to-base mutation rate r. We set p=0.01 and use a grid search to tune the hypermutation rate parameter r and the shape parameter α of the Gamma distribution to reflect the Parsimony Diversity observed in real data.

The goal is to match the PD of the simulated data to that of the real data. However, PD tends to increase with the number of unique sequences in the dataset. Therefore, we compare the PD of the data simulated on an UShER subtree to the dataset consisting of the observed sequences in that subtree. Still, the simulated dataset could differ in size from the corresponding real one, since we only use unique sequences. To correct for this, we also divide the number of nodes inferred on each dataset by the size of the dataset before comparing the two. We call this metric Corrected Increase in Parsimony Diversity (CIPD) and define it as

(1) $$CIPD=\frac{\;\text{Simulation}\;\text{PD}}{\;\text{Real}\;\text{data}\;\text{PD}\;}\cdot \frac{\;\text{Number}\;\text{of}\;\text{tips}\;\text{in}\;\text{real}\;\text{data}}{\;\text{Number}\;\text{of}\;\text{tips}\;\text{in}\;\text{simulation}}.$$

Compared to real data, simulated data have more MP groupings of taxa per taxon when CIPD is larger than 1, and fewer when CIPD is less than 1. When CIPD is exactly 1, that means the simulated data and real data admit similarly diverse sets of MP trees.

We use CIPD as a proxy for the relative increase in difficulty between real and simulated data. Previous work that simulates data in the densely sampled regime [7] does not evaluate the inferential difficulty of their simulated data, and our analysis in Section VII shows that the PD of their simulated data was on average lower than the PD of real data. In our work, we use a nearly identical simulation procedure, but tune the parameters in our simulation to make the inference problem sufficiently difficult.

#### Tuning simulation hyperparameters:

In total, for each of the 16 parameter combinations, we extracted 8 different UShER subtrees. For each subtree, we conducted 5 trials, where each trial consisted of randomly resolving the subtree, simulating sequences along its branches with phastSim [9], and computing the CIPD for that trial. We compute the CIPD by producing a large collection of MP trees stored in the history sDAG, as described in Section II-C, then finding all the unique clades among histories represented in the history sDAG. This can be done efficiently by looking at each node v=(ℓ,U) and storing the union of child clades $\cup_{C\in U}C$ in a set. Note that it is unlikely that our history sDAG contains all MP trees for the simulated or real datasets, thus we are underestimating the PD for both. However, since we have no reason to think that this error will be larger for real data than simulated (or vice versa), we expect this procedure generates a reasonable estimate for CIPD. We find that with α=0.1 and r=50 the simulation produces a realistic CIPD of 0.97. The median CIPD value for each combination of α and r are shown in Figure S3.

## III. Results

### A. Identifying subparsimonious structures in trees

When we simulate realistic sequence data using the methods in Section II-D, we find that the generative tree (topology) t is rarely MP. Let us refer to the trees contained in the trimmed history sDAG (Section II-C) as *inferred-MP trees*. Despite being subparsimonious, t often shares many clades with these inferred-MP trees, and the ways in which t deviates from them are through local simple structures.

**Fig. 2:**
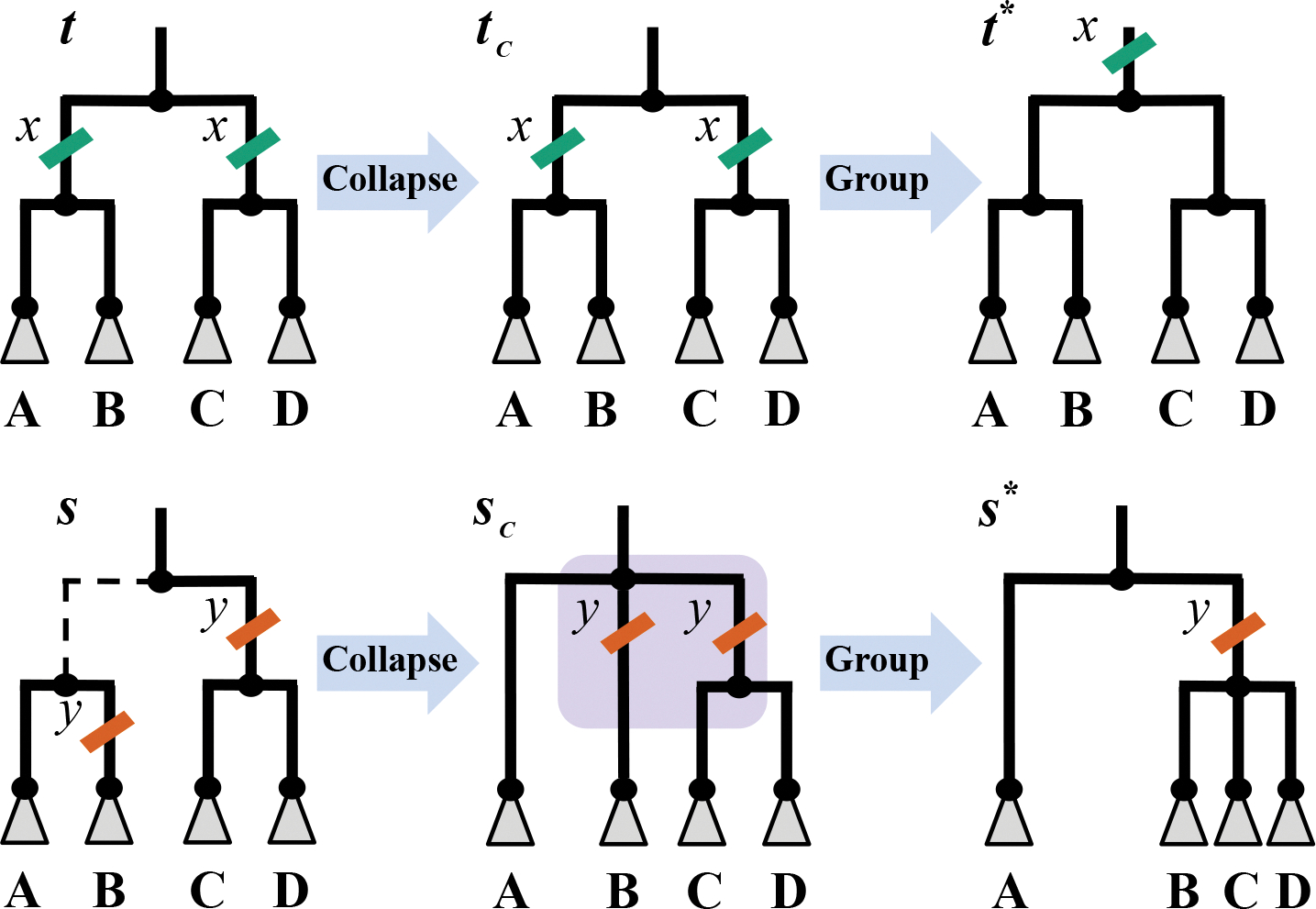
PCMs are locally duplicated mutations. Two examples of PCMs occurring in simulated trees t and s, and the corresponding most similar MP trees $t^{*}$ and $s^{*}$, are shown. The PCM in s causes a structural difference from the MP tree $s^{*}$. A PCM is detected by examining the collapsed simulated trees $t_{c}$ and $s_{c}$ for parallel child mutations on sister branches.

The most common type of local structural difference results from mutation patterns we call parallel child mutations (PCMs). A PCM is a subset of mutations that occur independently on two or more sister branches when a tree is collapsed to include only branches with mutations. The idea is that a PCM is a feature of a history representing independent, convergent evolution on nearby branches. PCMs are by definition subparsimonious, but they do not always induce subparsimonious structure. For example, consider the top example in Figure 2. The subset of the true phylogenetic history t is on the left and the subset of the history we infer using the MP criterion $t^{*}$ is on the right. The green tick indicates a set of mutations x that the taxa in subhistories A, B, C, and D all share. To make the simulated history more parsimonious, we can replace the two mutations with a single one on the branch corresponding to the clade A∪B∪C∪D, as in $t^{*}$. However, this does not change the tree topology itself.

We are more interested in situations where PCMs create subparsimonious *topology*. For example, consider the bottom example in Figure 2. The subset of the true phylogenetic history s is on the left and the subset of the history we infer using the MP criterion $s^{*}$ is on the right. In this case, the orange tick indicates a mutation shared by B, C, and D. When s is collapsed to $s_{c}$ by removing the dashed branch, and B, C, and D are grouped under a single branch, the topology changes. Specifically, $s^{*}$ contains the split A∣B,C,D, which is incompatible with the split A,B∣C,D in s. We observe that simulated trees have many topologically subparsimonious PCM-induced structures like those in s, but are globally quite similar to an MP tree.

**Fig. 3:**
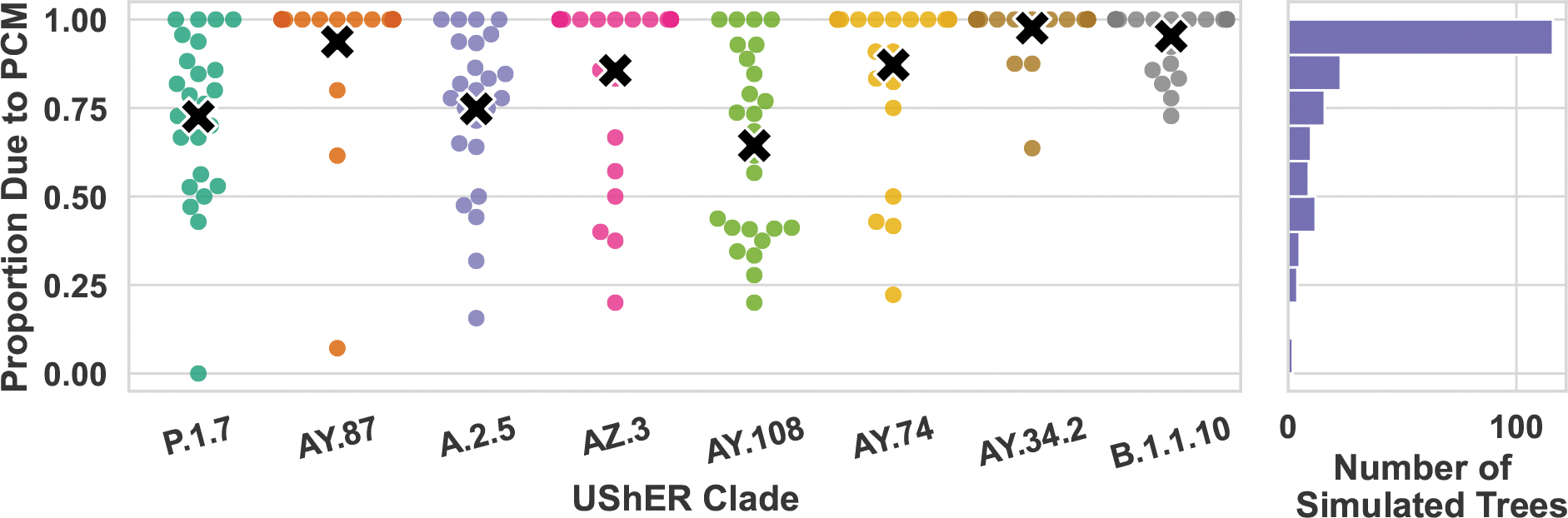
For simulations based on each UShER-clade, the swarm plot on the left shows the proportion of nodes in an MP tree that are missing in the corresponding true tree because of a PCM. The black X denotes the average within each clade. Some points on the y=1 line are overlapping, so the histogram shows the number of simulations with a particular proportion of differing nodes that can be explained by PCMs.

Examining all 200 simulated trees programmatically, we find that in at least 53.5% of the trials the *entire, tree-wide* difference between simulated tree and the nearest inferred-MP tree could be explained by PCMs (see Supplementary Materials Section V for further method details). Figure 3 breaks down the proportion of differing nodes that are due to a PCM by UShER-clade. The UShER-clade used to seed the simulation significantly affects this proportion. For clade AY.108, this number is as low as 60.6%, however for many clades it’s over 80%. Across all simulations, 1693 differing nodes out of the 2270 total (74.6%) could be explained by PCMs. These proportions are likely underestimates, since there may exist trees of the same parsimony score that are more similar to simulated trees, which we failed to find in our MP tree search. Later, we show how this insight can be used to estimate clade supports by modifying trees from the history sDAG to include these subparsimonious structures.

### B. PCM frequency increases with mutation rate

We hypothesized that the frequency with which a substitution is involved in a PCM increases with the mutation rate at the substitution site and target base. After all, instances of these high-rate substitutions will happen more frequently, and thus are more likely to occur independently on sister branches in PCMs. Getting a better understanding of this relationship could help us predict the frequency of PCMs in real examples, and potentially inform the design of phylogenetic algorithms that leverage this information.

**Fig. 4:**
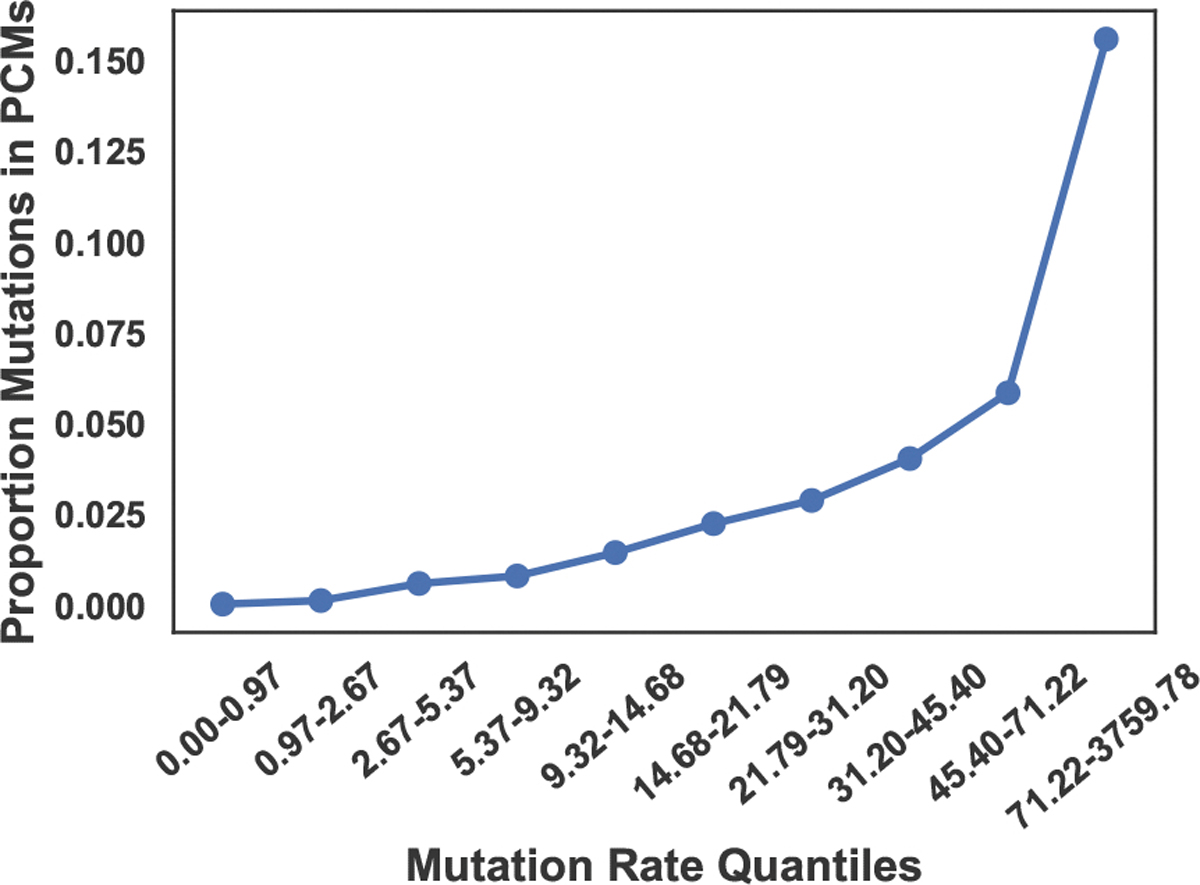
The pointplot shows the relationship between PCM proportion and mutation rate. The x-axis shows mutation-quantile buckets that contain mutations whose rate is within a given interval. The intervals were selected so that each contains 10% of all the mutations that occur across all 400 simulations. The y-axis is the proportion of mutations in a given bucket that were a part of a PCM.

Indeed, when we empirically examine this relationship in our simulations, we find that there is a clear increasing relationship (Figure 4). To perform this analysis, we summarized all substitutions that occurred in the simulations, along with their mutation rates and whether they are part of a PCM. Then, we selected decile intervals of mutation rate and plotted the average number of mutations within each interval. The frequency of PCMs steadily increases with mutation rate for the first nine deciles. No mutations with a mutability between 0 and 1 occur in PCMs, while over 5% of the mutations with a rate in the 45 to 70 range occur in PCMs. From the ninth to the tenth decile there is a sharp increase by almost 10%, which we suspect is due to the fact that the last decile contains mutations with much larger mutation rates.

### C. Estimating clade support from MP trees

One useful output of many phylogenetic algorithms is a measure of confidence in particular groupings of observed taxa in the inferred tree. This *clade support* measurement provides context for an interpretation of the inferred tree, such as an observation about the number of introductions that seeded an outbreak in a region. Often, clade support is computed by sampling many trees from a posterior distribution over trees using, e.g., Markov chain Monte Carlo (MCMC) and returning the proportion of samples that contain the given clade [11], [12]. In this case, a clade’s support can be interpreted as the probability that that clade is contained in the true tree under the specific evolutionary model assumptions. However, running MCMC can be prohibitively slow for large sequence alignments. Another approach is the bootstrap, which resamples the columns of the sequence alignment and then infers a tree on each resampled alignment [13]. This approach is less attractive in the case of densely-sampled viral data, where a given true edge may be supported by only one or a few mutations [14]. In this paper, we present two alternative support estimators that leverage the set of MP trees and can be computed quickly using the history sDAG.

To explore our observation that PCMs are the primary reason that realistic trees fail to be maximally parsimonious, we estimate clade support by sampling from two simplified distributions. The first distribution is uniform on all inferred-MP trees, and the second is more diffuse, introducing PCMs to inferred-MP trees during the sampling process.

#### Uniform MP posterior:

As a first attempt (to be improved upon below) at estimating clade support from collections of highly parsimonious histories, one can make the simplifying assumption that the posterior is uniform among the MP histories (Definition 1). After all, in MP inference these histories are deemed equally optimal, and we have no reason to expect any individual one to be more likely than the rest. In that case, the support for a clade is simply the proportion of MP histories which contain that clade.

To estimate this quantity, we build a large history sDAG (Section II-C), trim it to express only the most parsimonious histories (Section II-B), and count the proportion of inferred-MP histories that contain a given clade. This last step can be done efficiently with a dynamic programming algorithm in the history sDAG. Note that this procedure only approximates the uniform MP posterior because the history sDAG will probably only contain a subset of the MP trees. Still, to our knowledge, this is the best way to produce a large set of highly parsimonious (and likely MP) trees.

A more fundamental limitation of this support estimator is that it relies on the assumption that non-MP trees have no probability. In our simulations, we find that the simulated tree is rarely MP. So, under this uniformity assumption, the simulated tree will always have zero probability, which seems highly undesirable.

#### MP-diffused posterior:

To account for the fact that non-MP histories should be assigned non-zero probability, we define a distribution that spreads out the probability mass on each inferred-MP history. We achieve this by sampling a history from the history sDAG, randomly perturbing it, and counting the proportion of resulting histories that contain the clade of interest.

Our random perturbation is designed to increase the likelihood of sampling histories with PCMs to reflect the insight that the inferred-MP histories differ from the simulated tree primarily by PCMs. To do this, we *collapse* edges according to the probability that their mutations occurred independently and resolve the resulting history (for an example see $s\to s_{c}$ in Figure 2). Specifically, for the set of mutations M on a given edge e we consider the probability that those mutations occurred independently. We sample a number of additional mutations n∼Binomial(|Ch(e)|,p), where Ch(e) is the set of child edges of $e,p=\prod_{m\in M}p_{m}$, and $p_{m}$ is the probability that mutation m occurred. We randomly select n edges among Ch(e) to collapse, and do so by removing them from their parent node, attaching them to the parent node of e, and adding M to their list of mutations. We repeat this probabilistic collapse step for each edge of the history in a pre-order traversal, and return a uniformly random fully resolved history that is compatible with the collapsed version. The result is a history sampled from a distribution that has non-zero probability of sampling histories with PCMs.

If there are no mutations on the edge, we set the probability of collapse to 1 to reflect the fact that there is no phylogenetic support for that edge being present in the generative tree. For edges with mutations, we find that setting the probability of each mutation to $p_{m}=0.02$ works well on our simulations. We observed that the mean of distribution of parsimony scores of histories sampled with this setting of $p_{m}$ was near the parsimony score of the simulated history for many simulated datasets. Of course, this cannot be done without knowledge of the true number of mutations. We expected that estimating $p_{m}$ from features of the dataset like parsimony diversity would yield similar support estimation results, and indeed see this in a preliminary implementation (results not shown).

#### Fast diffused support sampling:

The two-step sampling procedure to estimate the MP-diffused posterior is very sample-inefficient. Indeed, we compute this estimator by sampling histories from the history sDAG, injecting PCMs, resolving multifurcating nodes uniformly, and adding one unit to a counter associated to each clade observed in the resulting tree. However, it is common for MP trees to include large multifurcations, where the data do not inform a choice between any of the many possible binary resolutions. Such a large multifurcation may need to be sampled, and resolved randomly, very many times for the resulting clade supports to approximate the true support, since many clades occur in only a tiny fraction of possible binary resolutions.

We can reduce the number of samples required, and sample more clades with a single MP tree chosen from the history sDAG, by avoiding taking a single choice of binary resolution. Instead, we can compute the fraction of binary resolutions containing each clade that could possibly result from a resolution of a multifurcating node. Instead of adding 1 to the counter for each resulting clade, we can add this fraction.

A multifurcating node with n children has b(n)=(2n−3)!! possible binary resolutions. To compute how many of these resolutions contains a particular monophyletic grouping of s<n children of the node, we need only multiply the number of binary arrangements of those n children, and the number of binary arrangements relating the remaining n−s leaves and the clade of size s. Therefore, there are b(s)×b(n−s+1) binary resolutions of a multifurcating node with n children, containing each possible child clade of size s. We can convert this to a fraction of possible binary resolutions by normalizing by b(n).

Since the set of clades which can result from resolving a multifurcation of size n scales as the powerset of n elements, it is impractical to iterate through each possible clade for large multifurcations. Instead, we ignore any clades whose frequency among all possible binary resolutions of the node would be less than a threshold of our choosing. This adjustment to our clade sampling method allows us to make more accurate estimates of clade supports with fewer samples of MP trees from the history sDAG.

### D. Support evaluation

We compare our MP-based support estimators to two traditional methods for support estimation: Bayesian posterior, implemented as MrBayes [11], and Bootstrap, implemented as Ultrafast Bootstrap (UFBoot2) [15]. For MrBayes, we used 10^8^ total iterations, with 7 · 10^7^ iterations of burnin, and sampled a tree once every 1000 iterations. We also used the most general GTR model with four categories of Gamma distributed rate variation. For UFBoot2, we used 1000 bootstrap replicates and allowed nearest neighbor interchange operations to further optimize bootstrapped trees [16].

**Fig. 5:**
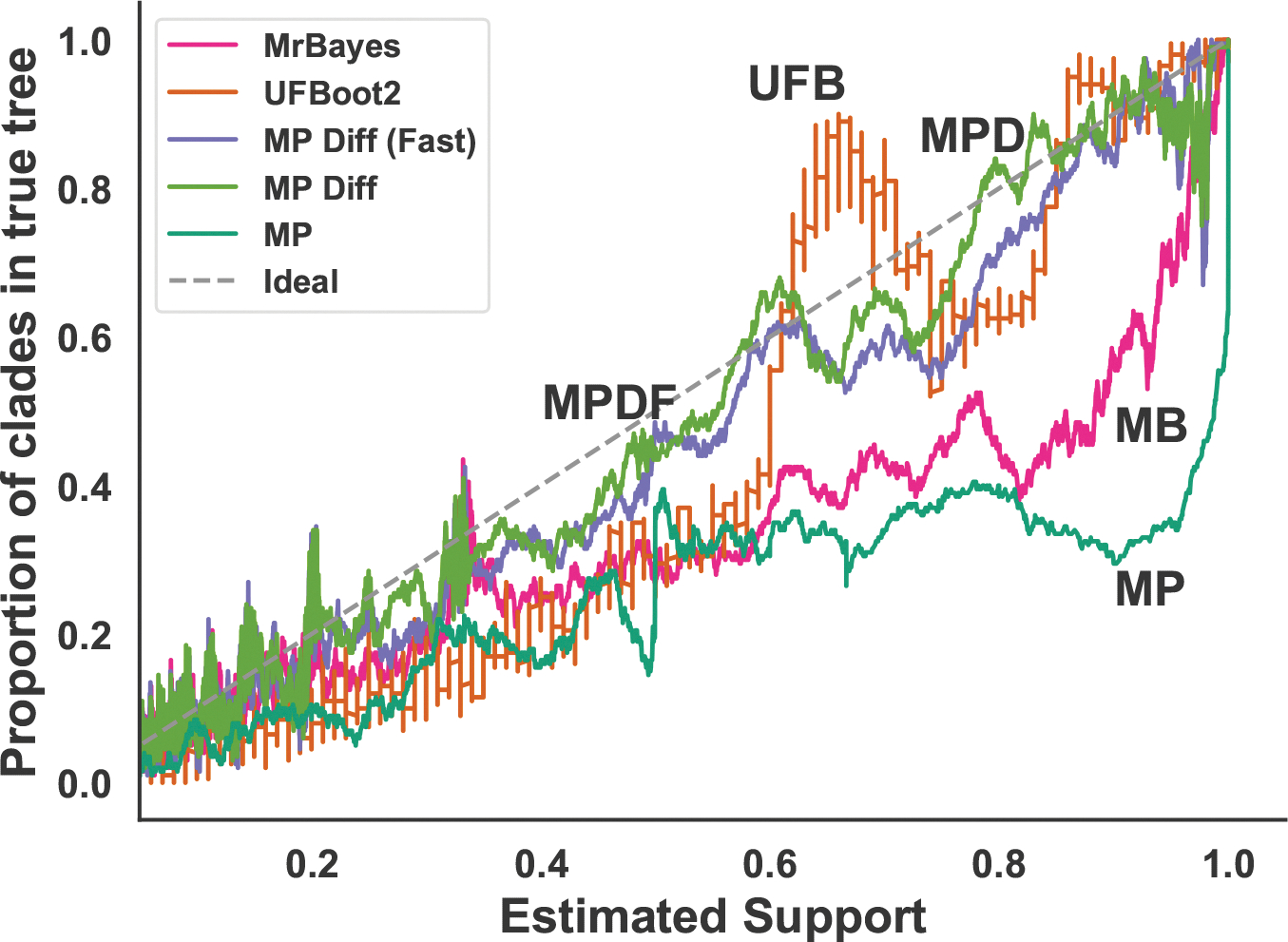
Calibration curves for each support estimation method: MrBayes, UFBoot2, MP, MP-Diffused, and MP-Diffused (Fast). Direct labels are MB, UFB, MP, MPD, and MPDF, respectively. The x-axis is the estimated support value, and the y-axis is the proportion of clades in the 200 length window that were in the true tree. For ease of plotting, we omit points with an estimated support in the range (0, 0.05), which does not affect the trend.

For each method, we plot a calibration curve by first computing the support values for data generated by the simulation procedure described in Section II-D. We then sort the clades (across all simulated datasets) by their estimated support value. For each estimated support value, we compute the empirical probability for that clade given its support value as the proportion of clades in a 200 length sliding window that are present in the generative tree. The calibration plot in Figure 5 summarizes these results.

In Figure 5, overestimation can be seen when the plotted curve is far to the right of the ideal y=x line. This indicates that the estimated support is greater than true support. The default MP support estimator consistently overestimates the supports values (i.e., for any given clade the estimated support is *higher* than its empirical support). MrBayes performs slightly better, but shows similar overestimation for estimated values in the range of 0.4 to 0.95. UFBoot2 also overestimates, but its curve is significantly closer to ideal than MrBayes or default MP support, especially for estimated supports in the range of 0.6 to 0.9. The calibration curves for both versions of MP-Diffused are closest to ideal for almost all predicted support values.

In addition to providing a potentially useful way of accurately estimating clade supports on densely sampled data, the success of the MP-Diffused clade support method provides further evidence that we can closely approximate the true distribution of trees conditioned on the data by adding PCMs to MP trees.

## IV. Conclusions

In summary, we have found that subparsimonious structures in simulated densely sampled phylogenies are often the result of a simple, local mutation duplication which we call a PCM. This observation allows us to estimate clade support by perturbing MP trees to include these subparsimonious structures. We find that this is a useful clade support estimator for simulations that are calibrated to mimic real densely-sampled data.

### A. Parsimony-suboptimal structures in simulations

Subparsimoniousness is a classical concept and many metrics have been proposed to measure it [17], [18]. One widely used approach for measuring subparsimoniousness is Consistency Index (CI), which measures the amount of homoplasy in a given tree by taking the ratio m/s, where m is the minimum number of character state changes to explain the observed sequences (i.e., the MP score) and s is the observed number of character state changes that occurred. A CI score of 1 means that the sequences evolved without homoplasy, and as the number of homoplasies goes up the CI goes down. This metric gives a sense of how close an evolutionary history is to being MP, but it does not shed any light on which substructures are subparsimonious and what mutation patterns give rise to them. In this work, we focus on these latter questions in a specific, yet highly relevant, parameter regime.

Despite the fact that homoplasy is a realistic feature of evolutionary histories, the MP criterion remains useful, particularly in the densely sampled regime where datasets are large and branch lengths are short. Theoretical work has also shown that the ML tree will be the same as the MP tree under simple models of evolution if the branch lengths are assumed to be very small [19]. More recent results show that in the near-perfect regime where tree length is small, it is likely that the splits in the (collapsed) MP tree are correct [1]. In our simulations, we confirm that the true tree can frequently be made maximally parsimonious by making local edits around the subparsimonious splits (e.g., collapsing short edges and grouping them under a common mutation).

We go further by showing that these subparsimonious splits are often PCMs (Section III-A), and propose a method (Section III-C) that uses this insight to perturb MP trees in more realistic ways. Although this approach produces fairly accurate estimates of clade support, the way in which it perturbs MP trees is very simple. When collapsing an edge, it only considers the number of mutations on that branch and ignores the types of mutations that occur on it. One line of future work could examine ways to make this form of perturbation even more realistic by, for example, taking the mutability of the mutations on a branch into account. Our insight that PCMs are much more frequent among mutations with high mutability (Section III-B) indicates that incorporating this information into the MP tree perturbation could further improve tree sampling.

### B. Comparison to other methods

Support estimates from diffused MP-sampling are well calibrated compared to the other approaches, and show the least overestimation. All five methods exhibit overestimation: the naive MP clade support estimate is the most overconfident, followed by MrBayes and UFBoot2, and the two MP-diffused estimates appear to be most accurate.

For the naive MP clade support and MrBayes methods, the overestimation of support is correlated with the assumptions each method makes about parsimony scores of realistic trees. The naive MP clade support method is based on the certainly incorrect assumption that all plausible trees explaining the data are MP. It will therefore place too much probability on the clades that are among MP trees, and overestimate their support.

One could argue for the use of Bayesian methods to infer phylogenetic relationships between densely sampled data since they allow for subparsimonious structures. However, we observe that a straightforward application of MrBayes, using the most general model settings available, samples trees that are also substantially more parsimonious than the simulated tree. In fact, if we compute the best possible parsimony score of any tree on the simulated data, we observe that the distribution of parsimony scores in MrBayes samples is often concentrated very near this best MP score. Figure S5 in the Appendix shows an example of this. Since a matching parsimony score is a necessary condition for two trees to have the same topology, this makes sampling the true tree with MrBayes extremely unlikely. In fairness to MrBayes, this behavior is certainly the result of a model misspecification, since the most general model available in MrBayes is not expressive enough to match our simulation model. However, since our simulations are designed to mimic real densely sampled data, we can expect a similar model misspecification, and corresponding overconfidence in clade support estimates, on real data.

Another challenge with Bayesian methods is that they are computationally intensive and cannot scale to extremely large sets of densely sampled data, such as that available for SARS-CoV-2. Recent work leverages parsimony to inform proposals that speed up MCMC chain convergence and improve mixing time [20]. We hope our results about subparsimonious structure, and our ability to sample realistic trees from a collection of MP trees, might motivate new proposal distributions for a Bayesian phylogenetic program.

UFBoot2 computes clade support estimates much faster than MrBayes. Also, the authors show empirically that UFBoot2 can yield high-accuracy estimates of clade-membership probability even in the presence of model violations and thus be interpreted as probabilities. However, Figure 5 shows that UFBoot2 clade support estimates do not match observed clade supports in our simulations. Moreover, unlike the other methods, the support values from UFBoot2 are not always overestimates. For support ranging from 0.6 to 1, the support values oscillate between overestimation and underestimation. Since the support values produced by UFBoot2 use ML trees on *bootstrapped sequences*, it is not clear how this estimate is related to a distribution of phylogenetic trees on the *real sequences*, and makes it difficult to discern the source of these inaccuracies.

### C. Importance of the history sDAG

Producing clade support estimates from MP trees would be extremely difficult without the history sDAG. Because parsimony diversity is often high on densely sampled, SARS-CoV-2-like data, any clade support estimate based on a single MP tree would likely be quite sensitive to *which* MP tree is chosen. The vast diversity of MP trees necessitates the use of an efficient data structure for searching for, storing, and sampling MP trees. The history sDAG is therefore essential for tuning simulation parameters using parsimony diversity and sampling from the large collection of MP trees, which is necessary to estimate clade support.

## Supplementary Material

1

## Data Availability

The SARS-CoV-2 data used to produce clade simulations in the Simulations section was read from the public SARS-CoV-2 tree distributed by the UShER team at http://hgdownload.soe.ucsc.edu/goldenPath/wuhCor1/UShER_SARS-CoV-2/. This data originates from GenBank [21] at https://www.ncbi.nlm.nih.gov, COG-UK [22] at https://www.cogconsortium.uk/tools-analysis/public-data-analysis-2/, and the China National Center for Bioinformation [23]–[26] at https://bigd.big.ac.cn/ncov/release_genome.

## Code Availability

The history sDAG data structure described in [5], as well as various algorithms described in this paper and in future work, are implemented in the open source Python package historydag, which is available at https://github.com/matsengrp/historydag. All code necessary to reproduce the SARS-CoV-2 simulations and analysis is available at https://github.com/matsengrp/hdag-benchmark.
