# Supplementary material for: The structure of deviations from maximum parsimony for densely-sampled data and applications for clade support estimation
Source: IEEE Trans Comput Biol Bioinform. Author manuscript; Available in PMC 2025 Nov 4. (PMC12582588; doi:10.1109/TCBBIO.2025.3595782)
Supplement: 1 [file NIHMS2119506-supplement-1.pdf]

Supplementary Materials

V. Identifying PCM-Induced Subparsimonious Clades

Denote the most similar MP tree as t^* and let C_t, C_{t^*} be the set of clades in t and t^*, respectively. Then, we define the clades $C = C_t \cap C_{t^*}$ in both trees as *jointly-MP with respect to* t^*, and the clades $C^{(t)} = C_t \setminus C$ in t not present in t^* as *jointly-subparsimonious with respect to* t^*. We pay special attention to the clades $C^{(t)}$ that have a corresponding clade in $C^{(t^*)}$ and systematically identify the differences between them. Studying these differences sheds light on the primary mechanism by which simulated trees are subparsimonious. To this end, our goals are to (i) find the largest set of clades in t that are jointly-MP, and (ii) understand why the clades in $C^{(t)}$ are jointly-subparsimonious with respect to t^*.

The first objective can be formulated as solving the following optimization problem: Let T_{MP} be the set of MP trees on the dataset X.

$$t^* = \arg\min_{t' \in T_{MP}} d(t, t') \tag{2}$$

where $d(t, t') = |C_t \triangle C_{t'}|$ is the RF distance between two trees. We approach this problem by producing a large set of MP histories $\hat{T}_{MP}$ represented by a history sDAG (V, E), as described in Section II-C, trim it using the weight function defined in Section V-A to express only the minimum distance histories $\hat{T}^*_{MP}$. If the minimizer is not unique, we trim the history sDAG further using **MinTrim** where our weight function is the summed sequence disimilarity between the simulated history and the histories in the history sDAG. This is the sum of the hamming distances between the sequences of nodes that correspond to a clade that is present in both histories, and can be decomposed as sum of edge weight functions like RF distance. Once we have our single most similar history $\hat{t}$, we address the second objective by mapping the clades of $C^{(t)}$ to similar clades in $C^{(\hat{t})}$, and analyzing the surrounding mutations to find the proportion of differing clades that are the result of small local deviations from an MP clade.

Specifically, we consider the collapsed versions of the trees, t_c and $\hat{t}_c$, and find all the clades in $\hat{t}_c$ but not in t_c. Then, for each differing clade $c \in C_{\hat{t}_c} \setminus C_{t_c}$, consider the node v that corresponds to clade c in $\hat{t}_c$. Denote the parent of that node as p and let c_p be the clade corresponding to the parent. If $c_p \in C_{t_c}$ then t_c has a node that corresponds to c_p as well. Let's denote this node p'. If the branches from p' have multiple of the same mutation m, then m is a PCM in t. To determine whether the PCM corresponds to a structural difference between t_c and $\hat{t}_c$, we check to see if grouping the branches that share m under a single node in t would recover the differing clade c. If so, then that implies the simulated tree t has at least one node that is the result of the mutations m evolving independently, and that clade is a subset of c_p. Thus, c is the result of the PCM m.

A. Minimizing Robinson-Foulds Distance in the History sDAG

Before we can compare simulated trees to their most similar MP counterparts, we first must be able to find such a similar MP tree. To do so, we employ an efficient trimming algorithm on the history sDAG, an innovation without which this paper would not be possible. We start with a history sDAG containing as many MP trees on the simulated data as we can find. Section II-C details how we produce this initial history sDAG.

Next, we find the trees in this history sDAG which minimize Robinson-Foulds (RF) distance to the simulated tree. Often, the history sDAG (V, E) represents a set of histories T so large that we could never hope to find the minimum distance tree by comparing them one-at-a-time. Recall from Section II-B that **MinTrim**(V, E, f) allows trimming with respect to a history weight function f that decomposes as a sum over edges. Here we show that RF distance to a given tree can be written in such a way, which implies that we can find the set of RF minimizing trees in $O(E)$ rather than $O(T)$. We prove this in the following lemma.

Lemma 1. *Let (V^*, E^*) be the history sDAG returned by **MinTrim**(V, E, f) with $f(e) = 1 - 2\left(\mathbb{1}_{C_t}(c_e)\right)$. Let (V', E') be the history sDAG containing the histories in (V, E) which minimize the RF distance to t. Then, $(V', E') = (V^*, E^*)$.*

Proof. It suffices to show that there is a function f such that $d(t, t') \propto \sum_{e \in t} f(e)$. Informally, we want to show that we can represent a shifted version of RF distance with an edge decomposition.

Recall, that the RF distance between two rooted topologies t and t' is defined as $|C_t \triangle C_{t'}|$, where $C_t \subset \mathcal{P}(X)$ is the set of clades below nodes in the tree t. Edges in a rooted tree are in bijection with clades via the assignment of each edge to the clade below its target node. For an edge e in the tree t' let the clade associated to e via this assignment be denoted c_e. Therefore, a shifted version of rooted RF distance can be decomposed as a sum over edges of an arbitrary tree t', with respect to a fixed reference tree t.

$$\begin{aligned}
d(t, t') &= |C_t \triangle C_{t'}| \\
&= |C_{t'} \setminus C_t| - |C_{t'} \cap C_t| + |C_t| \\
&= \sum_{c \in C_{t'}} \mathbb{1}_{C_t^{\sim}}(c) - \sum_{c \in C_{t'}} \mathbb{1}_{C_t}(c) + |C_t| \\
&= |C_t| + \sum_{e \in t'} 1 - 2\left(\mathbb{1}_{C_t}(c_e)\right)
\end{aligned}$$

Since $|C_t|$ is constant with respect to the trees in the history sDAG, the tree(s) that minimize $d(t, t') - |C_t|$ also minimize $d(t, t')$. □

B. Trimming

Here we present pseudocode for **MinTrim** that was suggested by Definitions 14 and 15, and Lemma 11, from the paper that defined the history sDAG [5]. Let (V, E) be a history sDAG that expresses the histories T, $f : E \to V$ be an edge weight, and $g : T \to \mathbb{R}$ such that for any history $(V', E') \in T$, $g(V', E') = \sum_{e \in E'} f(e)$. The minimum weight of a subhistory below a given node $v = (\ell, U)$ is recursively defined as

$$M_f(v) = \sum_{C \in U} M_f(v, C)$$
$$M_f(v, C) = \min_{v_c \in \text{Ch}(v,C)} M_f(v_c) + f(v, v_c)$$

and $M_f(v) = 0$ if v is a leaf node.

We precompute $M_f(v, C)$ and $M_f(v)$ with a single a post-order traversal, which requires visiting each edge once, and store them as maps. Denote this operation, **AnnotateMinWeight**(V, E). The full trimming algorithm is presented below.

MinTrim$\big((V, E), f\big)$: Computes the minimum trim of a given a history sDAG (V, E) with respect to edge weight function f.

```
E* = {}, V* = {}
M_f(v), M_f(v, C) ← AnnotateMinWeight(V, E)
for edge e = (v, v_c) of E in post-order do
    if M_f(v_c) + f(v, v_c) = M_f(v, C(v_c)) then
        add e to E*
    end if
end for
for edge e = (v, v_c) of E in pre-order do
    add v, v_c to V*
end for
for edge e = (v, v_c) of E in post-order do
    if v ∉ V* or v_c ∉ V* then
        remove e from E*
    end if
end for
return (V*, E*)
```

This algorithm runs in $O(E)$, and by construction, **MinTrim** finds (V^*, E^*) such that

$$E' = \{(v, v_c) \in E \mid M_f(v_c) + f(v, v_c) = M_f(v, C(v_c))\},$$
$$V^* = \{v \in V \mid v \textit{ reachable from a path in } E'\}, \textit{and}$$
$$E^* = \{(v, v_c) \in E' \mid v, v_c \in V^*\}.$$

Lemma 11 of [5] proves that (V^*, E^*) does indeed contain the minimum weight histories.

C. MinTrim Speedup

We run **MinTrim** on history sDAGs (described in Section II-C) built from our simulated data (described in Section II-D) and compare the run time to naively searching for the minimum distance MP tree to the simulated one. **Naive** finds the minimizing tree by iterating through each history in the history sDAG one at a time comparing the RF distance between that tree and the simulated tree. As expected, this approach will take linear time in the number of histories contained in the history sDAG. We observe that in practice **MinTrim** runs significantly faster, especially when the history sDAG contains many histories (Figure S1). For example, when there are $\approx 10^9$ histories, **MinTrim** computes the minimum in less than 10 minutes while **Naive** takes almost a day and a half.

Note that the runtime of **MinTrim** is a function of the number of edges in the history sDAG, not necessarily the number of histories. This is why we see variation in the runtime for the same number of histories in Figure S1. In the worst case, when trees have no overlapping edges, **Naive** and **MinTrim** will have the same runtime. However, typically many trees share many of the same edges. **MinTrim** takes advantage of this to find the minimum distance maximum parsimony tree much more efficiently than would otherwise be possible without such a compact data structure as the history sDAG.

D. Branch-Length Sensitivity

We stress that our main result that PCMs compose the primary structural deviation between the true tree and an MP tree *only applies in the densely sampled regime*. To examine the importance of the small-branch assumption, we repeat the PCM-proportion experiment described in Section III-A for the AY.34.2 clade by performing 5 independent simulations on the same trees, but with branch lengths multiplied by 1, 2, 4, and 8 (Fig S2). For the original branch lengths, the median proportion of differing nodes that can be explained by PCMs is 81.8%. As we increase the branch lengths by a factor of 2, then 4, then 8, the median proportion decreases all the way to 52.6%. Based on these results, it appears that for less densely sampled data (i.e., trees with longer branches), the true tree's deviation from the set of MP trees is less explainable by PCMs.

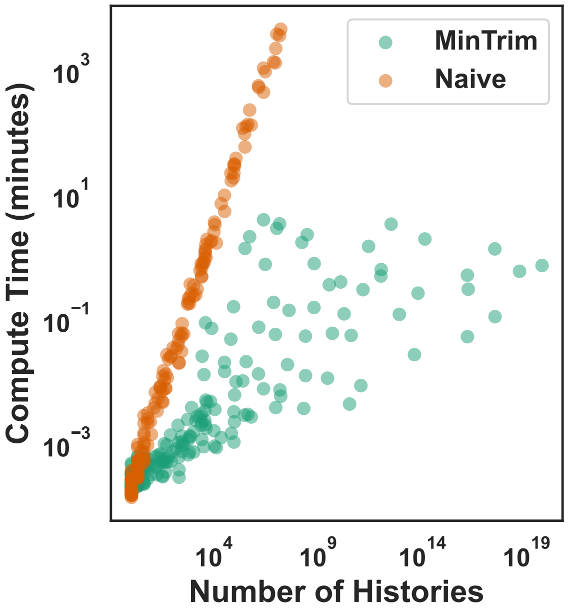

Fig. S1: Time in minutes to run **MinTrim** versus the naive approach on the history sDAG, which finds the minimum distance tree by comparing histories to the reference tree one at a time. The naive approach was cut off after two days.

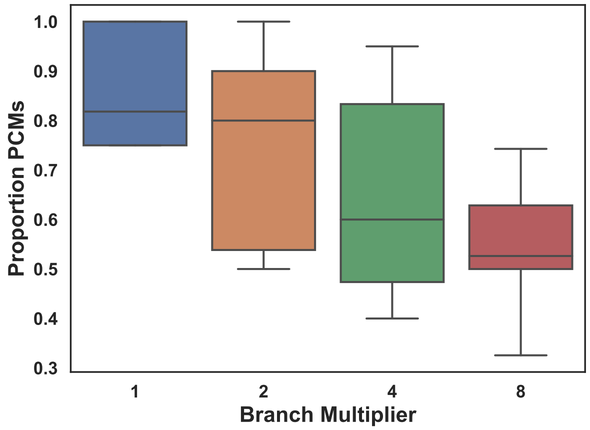

Fig. S2: Proportion of differences due to PCMs for various branch length multipliers. Each boxplot is taken over 5 independent trials.

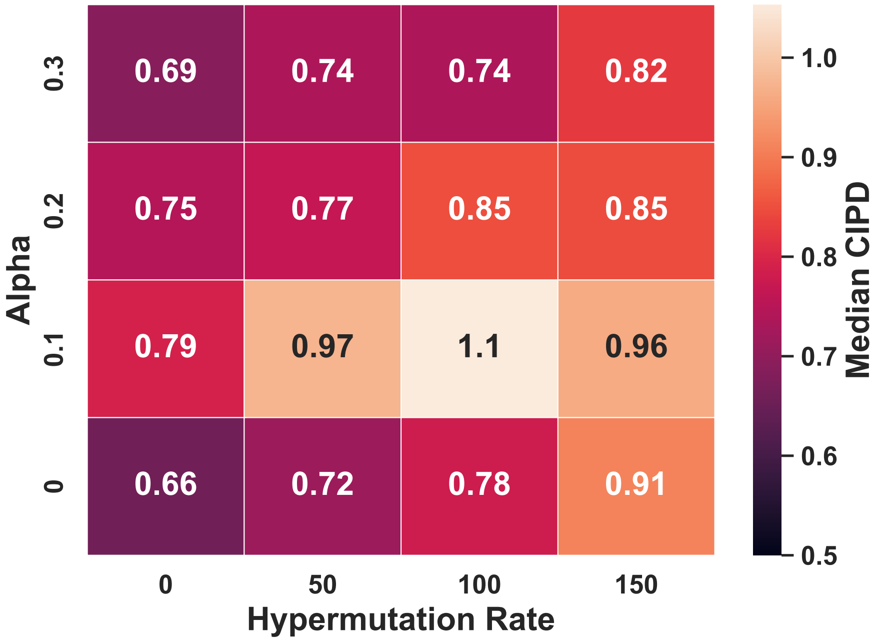

Fig. S3: Heatmap of median CIPD for each combination of α and r across all simulated datasets. When $\alpha = 0$ or $r = 0$, that indicates that we do not include the respective form of rate variation. When the CIPD is 1, the PD of simulated data matches that of the corresponding real data.

VI. Tuning Simulation Hyperparameters

Simulation parameters $(\alpha = 0.1, r = 50)$ yield among the best median CIPD values across all simulations (Figure S3). In addition to having a good median CIPD, we found that the variance in CIPD among simulations with $\alpha = 0.1, r = 50$ was lower than that of the other good parameter choices $(\alpha = 0.1, r = 100)$ and $(\alpha = 0.1, r = 150)$.

VII. CIPD for MAPLE Simulated Data

In [7], the authors benchmark their likelihood-based phylogenetic inference software by extracting subtrees from the UShER global tree and simulating mutations on that topology using phastSim [9] under three classes of simulation:

- The "basic" simulation scenario uses the GTR model with no rate variation and full genomes available.
- The "rate variation" scenario uses the GTR+G model with four genome site categories, all with the same frequency and with relative substitution rates of 0.1, 0.5, 1 and 2.
- The "sequence ambiguity" scenario modifies the basic scenario to include ambiguous characters.

We analyze the CIPD under similar settings for the second type of simulation. Specifically, we repeat the simulations described in Section II-D including topology selection and simulation with phastSim, but use categorical Gamma distributed rate variation and no hypermutation. Additionally, we use the UNREST substitution model instead of GTR. CIPD is computed as described in Section II-D.

We find that the CIPD values of the MAPLE datasets are significantly below 1 (Figure S4), indicating unrealistic levels phylogenetic inferential difficulty. All the simulations had a CIPD below 90% and half of them had a CIPD value below 70%. While some of our simulations have significantly higher CIPD than 1 (Figure S4), our distribution of CIPD is centered at 1 and almost all of our simulations have a CIPD value that differs from the ideal value, by no more than a factor of 2. It appears that hypermutation is important for achieving realistic levels of PD. We see further evidence of this in Figure S3 where all types of simulations without hypermutation (left-most column of heatmap) have a median CIPD less than 0.8. While we did not investigate the PD realism of the "basic" and "sequence ambiguity" simulations from [7], we expect these types of simulations to have CIPD values that are at least as small as the "rate variation" scenario because they do not include rate variation. We do however note that in [7], the authors simulate sequences on *much larger* UShER-clades than the ones

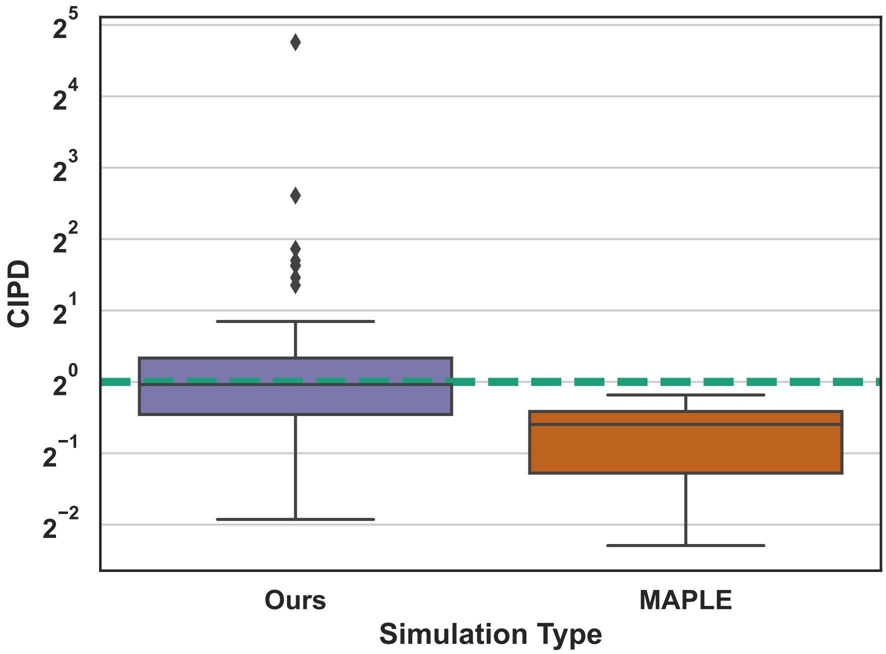

Fig. S4: The boxplots show CIPD values across all 200 simulations. The left boxplot uses our simulation settings with hypermutation at a rate of $r = 50$ and Gamma distributed rates using $\alpha = 0.1$. The right boxplot simulates without hypermutation and relative substitution rates of 0.1, 0.5, 1 and 2. The green dashed line indicates the CIPD value of simulated data with realistic PD.

that we use (i.e., between 2,000-20,000 tips). So, our analysis might not generalize to trees of that size.

Creating accurate and fair simulations is challenging, and the best method depends on the goal. In our work, we focus on simulating as realistic of data as possible because that is essential to generalizing our conclusions to real data sets. However, in [7], the authors use their simulations to compare their model's performance to other likelihood-based methods. They chose to not use more realistic simulations out of fairness since the other methods don't include UNREST and typically use GTR with four categories for rate variation.

VIII. MrBayes Parsimony Posterior

We simulate data as described in Section II-D and run MrBayes on the resulting set of sequences with the most general model settings available. This includes using the GTR substitution model and Gamma distributed rate variation.

In Figure S5, the red line shows the best parsimony score of the tree that the sequences were simulated on, and the histogram shows the distribution of parsimony scores in trees sampled from the MrBayes posterior. The MrBayes samples are significantly more parsimonious than the simulated tree. We suspect that this occurs because our inference model assumptions don't match the model under which we're simulating data. One example of this is that we include hypermutation in our simulations, whereas MrBayes doesn't model hypermutation. However, hypermutation appears to be a realistic feature of the data, so given that MrBayes infers a posterior that puts too much weight on high parsimony trees in these simulations, we expect these issues to carry over to real data as well.

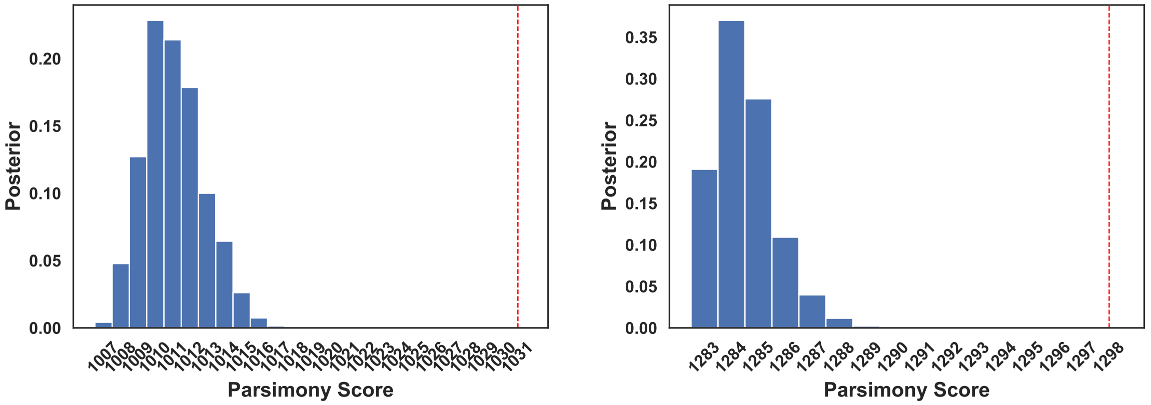

Fig. S5: Histogram showing the distribution of parsimony scores of topologies sampled with MrBayes, compared to the best possible parsimony score of the simulated topology, shown by the red dotted line. The topologies used for these simulations correspond to the subtree of UShER-clades AY.108 and P.1.7.
